# Machine learning model based on routine blood and biochemical parameters for early diagnosis of diabetic kidney disease

**DOI:** 10.3389/fendo.2026.1720574

**Published:** 2026-01-28

**Authors:** Wei Yong, Dan-dan Peng, Kai Ye, Jun-jie Gao, Ruo-xue Cao

**Affiliations:** 1 Department of Clinical Laboratory, The Second Affiliated Hospital of Wannan Medical College, Wuhu, China; 2 Department of Laboratory Medicine, The Second People’s Hospital of Lianyungang, Lianyungang, China; 3 Department of Laboratory Medicine, The Second People’s Hospital of Lianyungang Affiliated with Kangda College of Nanjing Medical University, Lianyungang, China

**Keywords:** diabetic kidney disease, early diagnosis, machine learning, risk prediction, routine blood parameters

## Abstract

**Background:**

Diabetic kidney disease (DKD) is the leading cause of end-stage renal disease globally, yet early diagnosis remains challenging due to conventional biomarker limitations, including UACR variability and reduced eGFR sensitivity. While machine learning shows promise in diabetes prediction, its application to early DKD identification using routine parameters remains underexplored. This study aimed to develop and validate machine learning models incorporating routine blood and biochemical parameters for early DKD prediction.

**Methods:**

This retrospective study analyzed 3,114 diabetic patients from the Second Affiliated Hospital of Wannan Medical College (EDN1) and 1,496 patients from NHANES 2005-2018 (EDN2) for external validation. Early DKD was defined as UACR 30–300 mg/g with eGFR ≥60 ml/min/1.73m². Seven machine learning algorithms were compared. Feature importance was assessed using SHAP framework, and Mendelian randomization explored causal relationships.

**Results:**

Among 3,114 patients, 1,333 (42.8%) had early DKD. Logistic regression achieved optimal performance (AUC = 0.689, sensitivity=40.5%, specificity=81.3%). Top predictors included triglyceride-glucose index (TyG), gender, creatinine, globulin, and age. External validation confirmed significant associations for HbA1c, globulin, TyG, and neutrophil-to-albumin ratio.

**Conclusions:**

The machine learning model successfully identified early DKD using routine parameters, with TyG index, HbA1c, and globulin as key predictors, demonstrating potential as a cost-effective screening tool.

## 1 Introduction

Diabetic kidney disease (DKD), the most common and severe microvascular complication of diabetes, has become the leading cause of end-stage renal disease (ESRD) worldwide (1). Its prevalence continues to rise alongside the global diabetes epidemic, imposing a substantial burden on patients’ quality of life and public health systems (2). Pathological changes in early-stage DKD exhibit a degree of reversibility; however, once the disease progresses to clinical proteinuria, renal function often deteriorates irreversibly (3). Therefore, early identification and intervention are crucial for delaying disease progression and improving prognosis (4).

Currently, the diagnosis of early-stage diabetic kidney disease in clinical practice relies primarily on the urine albumin-to-creatinine ratio (UACR) and estimated glomerular filtration rate (eGFR) (5). Nevertheless, UACR is subject to marked fluctuations throughout the day and can be affected by various confounders such as infections, physical activity, and changes in blood pressure. At the same time, eGFR shows limited sensitivity in identifying early or mild kidney dysfunction, which increases the likelihood of missed diagnoses in the initial stages of DKD. In addition, these assessments are not widely adopted in primary healthcare or routine physical check-ups, causing many high-risk patients to lose the chance for early detection (6). Recent studies indicate that DKD development is closely associated with systemic metabolic disorders and chronic inflammation (7). Routine blood and biochemical markers—including complete blood count, liver and kidney function, and lipid profiles—contain critical information about metabolic and inflammatory states (8). Composite indices derived from these markers, such as the triglyceride-glucose index (TyG), systemic immune-inflammation index (SII), and neutrophil-to-albumin ratio (NPAR), have been demonstrated to associate closely with insulin resistance and cardiovascular risk (9). However, their systematic application in comprehensive prediction of early DKD remains to be further explored (10).

With the rapid advancement of medical big data and artificial intelligence technologies, machine learning (ML) has emerged as a powerful tool for uncovering hidden patterns within high-dimensional, nonlinear clinical data (11). In diabetes research, ML models have been successfully applied to disease risk stratification and early diagnosis, even identifying individuals with normal fasting blood glucose levels who harbor diabetes, demonstrating predictive performance superior to traditional methods (12). However, existing research has focused primarily on predicting diabetes itself or its late-stage complications (13). Studies developing ML prediction models for early-stage diabetic kidney disease based on widely accessible routine examination indicators remain relatively scarce (14). Furthermore, most models lack independent external validation, limiting their clinical implementation and application.

**Figure 1:**
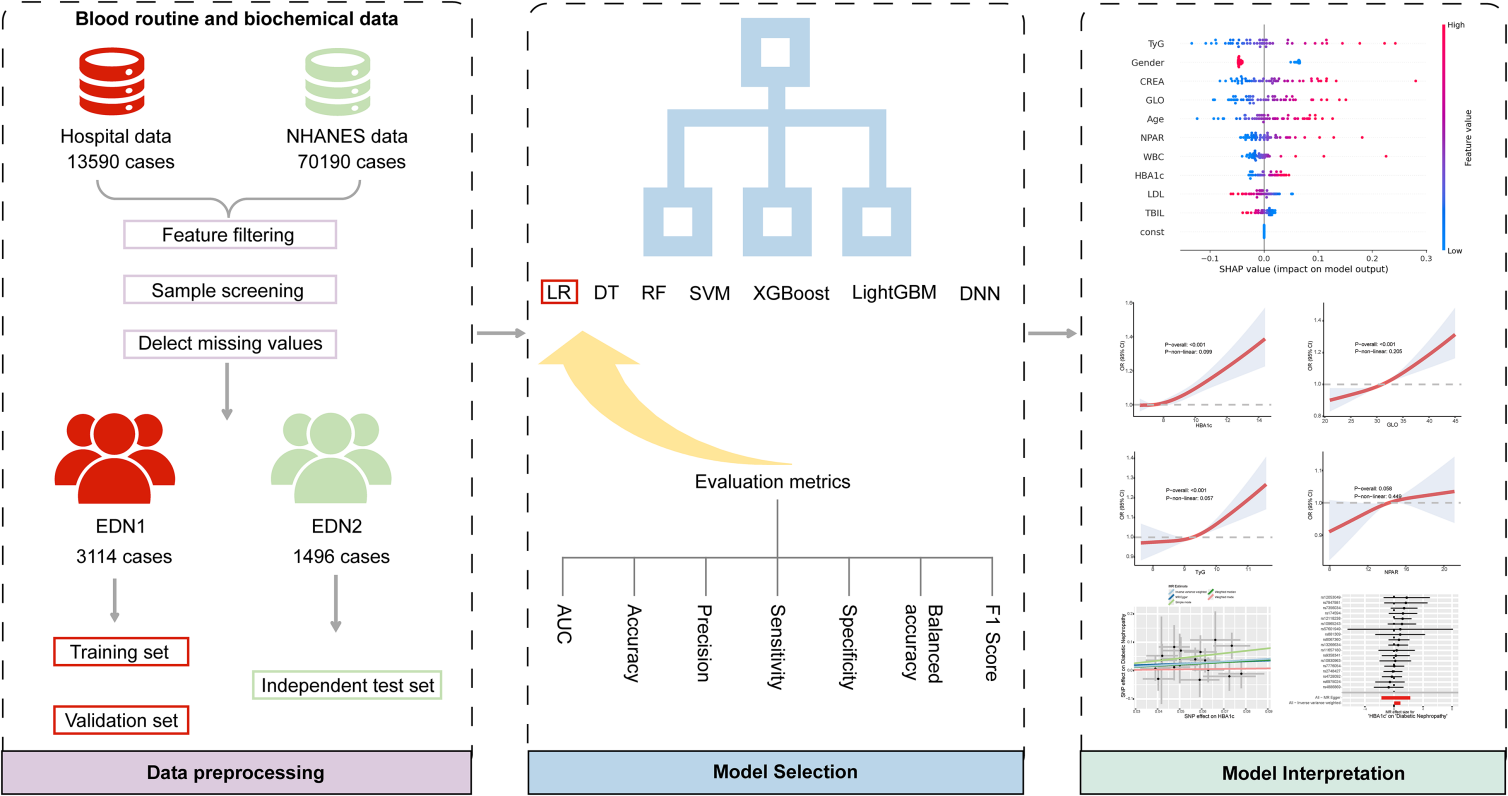
Complete analysis flow chart.

To address this research gap, this study integrates 55 conventional blood and biochemical indicators (including baseline markers and inflammatory-metabolic composite indicators). Utilizing multiple machine learning algorithms such as logistic regression (LR), random forest (RF), and XGBoost, we constructed a model for identifying early-stage DKD (defined as UACR 30–300 mg/g and eGFR ≥ 60 ml/min/1.73m²). By systematically comparing algorithm performance, we selected the optimal predictive model. Feature importance analysis and model interpretability were conducted using the SHAP (Shapley Additive Explanations) framework. External validation using the National Health and Nutrition Examination Survey (NHANES) database assessed generalization capability. Furthermore, Mendelian randomization (MR) analysis explored causal relationships between potential risk factors and DKD, providing genetic evidence for risk mechanisms. This study aims to develop an efficient, cost-effective, and clinically scalable tool for early DKD diagnosis, offering novel strategies for early screening and personalized risk management in diabetic nephropathy. The complete analysis process is shown in **Figure 1**.

## 2 Methods

### 2.1 Data collection and processing

Retrospective study data were sourced from The Second Affiliated Hospital of Wannan Medical College, while external independent validation data were obtained from the NHANES database covering 2005–2018. The two datasets were designated as EDN1 and EDN2, respectively. The initial step involved data cleaning to exclude samples with missing values and outliers. Diabetic patients with HbA1c ≥ 6.5% were identified based on WHO criteria for diabetes. Early diabetic nephropathy was defined as eGFR ≥ 60 ml/min/1.73m² and 30 ≤UACR <300 mg/g, while the simple diabetes group was defined as eGFR ≥ 60 ml/min/1.73m² and UACR < 30 mg/g. After preprocessing, 3,114 and 1,496 samples were retained for EDN1 and EDN2, respectively. The dataset was first split into training (70%, n=2,180) and validation (30%, n=934) sets using stratified random sampling to maintain class proportions. All hyperparameter tuning was performed exclusively within the training set using 5-fold stratified cross-validation. The final model was selected based on mean AUC across the 5 folds, then retrained on the entire training set with optimal hyperparameters before evaluation on the held-out validation set. This nested approach prevents information leakage and provides unbiased performance estimates. Subsequently, EDN1 was split into training and validation sets using a 7:3 ratio via stratified random sampling, while EDN2 served as an independent test set. While an *a priori* sample size calculation was not performed due to the retrospective study design, *post-hoc* analysis confirmed adequate statistical power. Following the ‘events per variable’ principle (minimum 10 EPV), our 1,333 early DKD cases with 10 predictors yielded 133 EPV, substantially exceeding recommendations (15). Additionally, recent machine learning guidelines suggest samples >1,000 are adequate for binary classification tasks (16), and our combined cohort (n=4,610) exceeds this threshold.

All datasets contained 55 clinical characteristics, including: Gender, Age, Glycated Hemoglobin (HbA1c), Alanine Aminotransferase (ALT), Aspartate Aminotransferase (AST), Albumin (ALB), Globulin (GLO), Alkaline Phosphatase (ALP), Total Bilirubin (TBIL), Direct Bilirubin (DBIL), Creatinine (CREA), Urea (UREA), Uric Acid (UA), Triglycerides (TG), Total Cholesterol (CHOL), Low-Density Lipoprotein Cholesterol (LDL-C), High-Density Lipoprotein Cholesterol (HDL-C), Gamma-Glutamyl Transpeptidase (GGT), Fasting Blood Glucose (GLU), White Blood Cell Count (WBC), Red Blood Cell Count (RBC), Hemoglobin (HGB), Platelet Count (PLT), Hematocrit (HCT), Mean Corpuscular Volume (MCV), Mean Corpuscular Hemoglobin (MCH), Mean Corpuscular Hemoglobin Concentration (MCHC), Neutrophil Percentage (NEUT%), Lymphocyte Percentage (LYMPH%), Monocyte Percentage (MONO%), Basophil Percentage (BASO%), Eosinophil Percentage (EO%), Neutrophil Absolute Count (NEUT#), Lymphocyte Absolute Count (LYMPH#), Monocyte Absolute Count (MONO#), Eosinophil Absolute Count (EO#), Basophil Absolute Count (BASO#), Red Cell Distribution Width (RDW), Triglyceride-Glucose Index (TyG), Systemic Immune Inflammation Index (SII), Systemic Inflammatory Response Index (SIRI), Neutrophil-Lymphocyte Ratio (NLR), Platelet-Lymphocyte Ratio (PLR), Neutrophil-High-Density Lipoprotein Ratio (NHR), Monocyte-to-High-Density Lipoprotein Ratio (MHR), Platelet-to-High-Density Lipoprotein Ratio (PHR), Lymphocyte-to-High-Density Lipoprotein Ratio (LHR), Neutrophil-to-Monocyte Ratio (NMR), Adaptive Immune Inflammation Index (AISI), Monocyte-to-Lymphocyte Ratio (MLR), Neutrophil-Albumin Ratio (NPAR), Neutrophil-High-Density Lipoprotein-Hemoglobin Ratio (NHHR), Uric Acid-High-Density Lipoprotein Ratio (UHR), Atherosclerosis Index (AIP), and Stress-Induced Hyperglycemia Ratio (SHR). The formulae for composite indicators are provided in (**Supplementary Table 1**). Outliers were identified using the interquartile range (IQR) method, where values exceeding Q3 + 3×IQR or below Q1 - 3×IQR were flagged. This conservative threshold (3×IQR rather than 1.5×IQR) was selected to retain clinically plausible extreme values while excluding likely data entry errors. Additionally, physiologically implausible values were manually reviewed (e.g., negative laboratory values, HbA1c >20%). Given the presence of categorical imbalance across all datasets, normalization was performed on all datasets.

### 2.2 Machine learning methods

Seven machine learning algorithms were implemented and compared:

Logistic Regression (LR) belongs to generalized linear models and is widely used for binary classification problems. This model estimates the probability of an event occurring through linear combinations and sigmoid function transformations, with output range (0,1) representing the likelihood of a sample belonging to a specific category.

Decision Tree (DT) is a nonparametric supervised learning method based on tree structure that recursively partitions data through a series of rules to perform classification tasks. Its model structure is intuitive and interpretable but sensitive to noise and prone to overfitting.

Random Forest (RF) is an ensemble learning algorithm that constructs multiple decision trees and integrates their predictions to enhance generalization ability and robustness. This algorithm effectively handles high-dimensional features and exhibits strong tolerance for outliers and noise.

Support Vector Machine (SVM) is a classification model based on the maximum margin principle, utilizing kernel functions to address non-linear problems. Its optimization objective is to find the optimal hyperplane that separates samples of different classes.

XGBoost (eXtreme Gradient Boosting) is an efficient machine learning algorithm based on the gradient boosting framework. It iteratively constructs a series of weak classifiers (decision trees) while optimizing the loss function to progressively reduce bias and variance.

LightGBM (Light Gradient Boosting Machine) is another efficient gradient boosting algorithm based on decision tree ensembles. It employs histogram-based optimization strategies and leaf growth policies to significantly accelerate training speed and reduce memory usage.

Deep Neural Networks (DNNs) are neural network models with multiple hidden layers that can automatically extract high-level feature representations through multi-layer nonlinear transformations.

To eliminate the impact of inconsistent feature scales on model performance, all continuous features underwent standardization. Hyperparameter tuning employed five-fold cross-validation combined with grid search and manual fine-tuning strategies. All hyperparameters were selected using the area under the curve (AUC) as the performance metric within the training set via 5-fold cross-validation.

### 2.3 Model performance evaluation

To comprehensively evaluate the performance of classification models, this study employs multiple widely recognized metrics, including sensitivity, specificity, balanced accuracy, and area under the receiver operating characteristic curve (AUC). Given the significant class imbalance between the validation and test sets, these metrics provide a more holistic reflection of the model’s overall discriminative capability across majority and minority classes. The calculation formulas for each metric are as follows. True positives (TP) represent the number of correctly predicted positive samples, false positives (FP) denote the number of negative samples incorrectly predicted as positive, true negatives (TN) indicate the number of correctly predicted negative samples, and false negatives (FN) signify the number of positive samples misclassified as negative. All metrics range from 0 to 1, with higher values indicating superior model performance.


$$\;\text{Accuracy}=\frac{\text{T}P+TN}{TP+FN+TN+FP}$$

$$\text{Precision}=\;\frac{TP}{\text{T}P+FP}$$

$$\;\text{Sensitivity}=\text{TPR}=\frac{\text{T}P}{\text{T}P+FN}$$

$$\text{Specificity}=\text{TNR}=\frac{TN}{\text{T}N+FP}$$

$$\text{Balanced accuracy}=\frac{TPR+TNR}{2}$$

$$\text{F}1\text{score}=\frac{2*(\text{Precision}*\text{TPR})}{\text{Precision}+TPR}$$

### 2.4 Model interpretation

To address the inherent “black box” nature of machine learning models, this study adopted the Shapley Additive Explanations (SHAP) framework based on cooperative game theory, providing a unified and interpretable method for quantifying feature importance. SHAP quantitatively assesses each feature’s marginal contribution to model output by assigning a SHAP value to each feature within each sample, thereby revealing the model’s decision-making mechanism at both individual and global levels.

### 2.5 Mendelian randomization analysis

To systematically evaluate the potential causal association between HbA1c levels and diabetic nephropathy, this study employed Mendelian randomization (MR) analysis using the TwoSampleMR software package (v0.5.6). Genetic instrumental variables were sourced from the GWAS database. Single nucleotide polymorphism (SNP) selection criteria included genome-wide significance (p <5×10^-8^), F-statistic >10 (to exclude weak instrument bias), and low linkage disequilibrium (r² <0.01). Causal effect estimates were derived using multiple methods to enhance robustness and reliability.

### 2.6 Statistical analysis

All statistical analyses were performed using R (version 4.4.3) and Python (version 3.10). The nonparametric Wilcoxon signed-rank test was employed to test for differences in characteristics between patient groups, with P <0.05 set as the threshold for statistical significance. Based on the finalized LR model, a nomogram model for individualized prediction was constructed using the rms R package.

## 3 Results

### 3.1 Baseline data results

We collected diabetes patient data from The Second Affiliated Hospital of Wannan Medical College between 2019 and 2025, enrolling a total of 3,114 eligible patients. These patients were categorized into those with uncomplicated diabetes and those with early diabetic nephropathy based on UACR criteria. (**Table 1**) describes the characteristics of individuals with and without early diabetic nephropathy, indicating that demographic and blood test indicators hold considerable potential for distinguishing early diabetic nephropathy patients within the diabetic population.

**Table 1:** Characteristics of patients with early diabetic nephropathy and those with diabetes mellitus alone.

Variables	Total (n = 3114)	Diabetes (n = 1781)	Diabetic nephropathy (n = 1333)	Statistic	*P*
Gender, n(%)				χ²=26.55	**<.001**
Female	1310 (42.07)	679 (38.12)	631 (47.34)		
male	1804 (57.93)	1102 (61.88)	702 (52.66)		
Age, Mean ± SD	59.84 ± 13.80	58.14 ± 13.25	62.11 ± 14.19	t=-7.94	**<.001**
HBA1c, Mean ± SD	9.56 ± 2.36	9.42 ± 2.33	9.75 ± 2.39	t=-3.86	**<.001**
ALT, Mean ± SD	27.11 ± 60.29	28.78 ± 72.30	24.88 ± 38.74	t=1.79	0.074
AST, Mean ± SD	26.20 ± 47.04	26.54 ± 56.50	25.74 ± 30.08	t=0.47	0.642
ALB, Mean ± SD	41.29 ± 4.08	41.53 ± 3.84	40.96 ± 4.38	t=3.78	**<.001**
GLO, Mean ± SD	29.35 ± 4.62	28.73 ± 4.31	30.18 ± 4.89	t=-8.58	**<.001**
ALP, Mean ± SD	80.61 ± 32.71	79.49 ± 29.31	82.10 ± 36.73	t=-2.20	**0.028**
TBIL, Mean ± SD	12.33 ± 5.95	12.81 ± 6.50	11.68 ± 5.06	t=5.48	**<.001**
DBIL, Mean ± SD	3.27 ± 2.32	3.37 ± 2.68	3.13 ± 1.71	t=2.82	**0.005**
CREA, Mean ± SD	74.17 ± 30.34	70.41 ± 24.11	79.19 ± 36.47	t=-7.63	**<.001**
UREA, Mean ± SD	6.00 ± 2.56	5.74 ± 1.91	6.34 ± 3.21	t=-6.12	**<.001**
UA, Mean ± SD	325.53 ± 99.16	318.76 ± 91.22	334.58 ± 108.23	t=-4.31	**<.001**
TG, Mean ± SD	2.03 ± 2.44	1.88 ± 2.01	2.22 ± 2.90	t=-3.67	**<.001**
CHOL, Mean ± SD	4.55 ± 1.27	4.57 ± 1.18	4.53 ± 1.38	t=0.87	0.382
LDL, Mean ± SD	2.68 ± 0.91	2.72 ± 0.89	2.62 ± 0.94	t=3.20	**0.001**
HDL, Mean ± SD	1.10 ± 0.34	1.10 ± 0.34	1.10 ± 0.35	t=-0.15	0.877
GGT, Mean ± SD	38.51 ± 52.34	37.71 ± 48.86	39.58 ± 56.65	t=-0.98	0.326
GLU, Mean ± SD	8.65 ± 3.73	8.39 ± 3.48	8.98 ± 4.02	t=-4.27	**<.001**
WBC, Mean ± SD	6.62 ± 2.54	6.38 ± 2.06	6.93 ± 3.05	t=-5.62	**<.001**
RBC, Mean ± SD	4.50 ± 0.61	4.54 ± 0.59	4.44 ± 0.64	t=4.69	**<.001**
PLT, Mean ± SD	192.03 ± 63.28	191.51 ± 60.80	192.72 ± 66.48	t=-0.52	0.605
HGB, Mean ± SD	135.39 ± 18.40	136.98 ± 17.75	133.25 ± 19.03	t=5.57	**<.001**
HCT, Mean ± SD	40.75 ± 5.03	41.23 ± 4.83	40.11 ± 5.22	t=6.13	**<.001**
MCV, Mean ± SD	90.93 ± 5.01	91.07 ± 5.01	90.74 ± 4.99	t=1.82	0.068
MCH, Mean ± SD	30.18 ± 2.03	30.23 ± 2.03	30.12 ± 2.03	t=1.55	0.121
MCHC, Mean ± SD	331.92 ± 13.35	331.92 ± 12.57	331.93 ± 14.33	t=-0.02	0.981
NEUT %, Mean ± SD	59.57 ± 10.38	58.26 ± 9.70	61.31 ± 10.99	t=-8.04	**<.001**
LYMPH %, Mean ± SD	30.29 ± 9.57	31.39 ± 9.01	28.81 ± 10.09	t=7.38	**<.001**
MONO %, Mean ± SD	7.32 ± 2.21	7.40 ± 2.18	7.21 ± 2.25	t=2.31	**0.021**
EO %, Mean ± SD	2.33 ± 1.93	2.42 ± 2.09	2.20 ± 1.68	t=3.27	**0.001**
BASO %, Mean ± SD	0.50 ± 0.28	0.53 ± 0.29	0.47 ± 0.28	t=5.08	**<.001**
NEUT #, Mean ± SD	4.06 ± 2.29	3.80 ± 1.76	4.41 ± 2.82	t=-7.05	**<.001**
LYMPH #, Mean ± SD	1.91 ± 0.70	1.95 ± 0.68	1.85 ± 0.71	t=3.59	**<.001**
MONO #, Mean ± SD	0.47 ± 0.23	0.46 ± 0.18	0.49 ± 0.29	t=-2.87	**0.004**
EO #, Mean ± SD	0.14 ± 0.12	0.15 ± 0.13	0.14 ± 0.11	t=1.91	0.057
BASO #, Mean ± SD	0.03 ± 0.02	0.03 ± 0.02	0.03 ± 0.02	t=2.67	**0.008**
RDW, Mean ± SD	12.73 ± 1.08	12.67 ± 1.07	12.81 ± 1.09	t=-3.59	**<.001**
TyG, Mean ± SD	9.18 ± 0.89	9.11 ± 0.85	9.26 ± 0.95	t=-4.41	**<.001**
SII, Mean ± SD	533.61 ± 1119.16	445.29 ± 588.74	651.62 ± 1561.95	t=-4.59	**<.001**
SIRI, Mean ± SD	1.45 ± 3.66	1.17 ± 2.90	1.81 ± 4.45	t=-4.56	**<.001**
NLR, Mean ± SD	2.65 ± 3.82	2.29 ± 2.71	3.13 ± 4.89	t=-5.66	**<.001**
PLR, Mean ± SD	114.76 ± 76.55	109.81 ± 59.81	121.39 ± 94.01	t=-3.94	**<.001**
NHR, Mean ± SD	4.22 ± 5.87	3.80 ± 2.32	4.79 ± 8.53	t=-4.14	**<.001**
MHR, Mean ± SD	0.49 ± 0.45	0.46 ± 0.25	0.52 ± 0.62	t=-3.06	**0.002**
PHR, Mean ± SD	192.79 ± 105.91	189.67 ± 85.77	196.95 ± 127.88	t=-1.80	0.072
LHR, Mean ± SD	1.92 ± 1.05	1.94 ± 0.98	1.89 ± 1.13	t=1.45	0.146
NMR, Mean ± SD	9.17 ± 6.06	8.72 ± 4.20	9.77 ± 7.86	t=-4.44	**<.001**
AISI, Mean ± SD	315.94 ± 1357.40	239.89 ± 724.99	417.55 ± 1893.59	t=-3.25	**0.001**
MLR, Mean ± SD	0.29 ± 0.24	0.27 ± 0.21	0.31 ± 0.27	t=-4.77	**<.001**
NPAR, Mean ± SD	14.63 ± 3.44	14.18 ± 2.96	15.24 ± 3.91	t=-8.34	**<.001**
NHHR, Mean ± SD	3.47 ± 2.05	3.45 ± 1.62	3.51 ± 2.51	t=-0.77	0.443
UHR, Mean ± SD	334.43 ± 242.34	321.13 ± 152.87	352.20 ± 324.76	t=-3.24	**0.001**
AIP, Mean ± SD	0.16 ± 0.36	0.14 ± 0.35	0.19 ± 0.38	t=-3.22	**0.001**
SHR, Mean ± SD	0.71 ± 0.27	0.70 ± 0.25	0.72 ± 0.30	t=-2.29	**0.022**

t, t-test; χ², Chi-square test.

SD, standard deviation. Bold font indicates p-value <0.05.

### 3.2 Regression analysis for screening diagnostic markers

To investigate the association between early diabetic nephropathy and multiple clinical indicators, this study employed univariate and multivariate binary logistic regression analysis (**Table 2**). Univariate analysis revealed that multiple variables—including gender (male vs. female, OR = 0.69), age (OR = 1.02), HbA1c, GLO, TBIL, CREA, LDL, WBC, TyG index, and NPAR—were significantly associated with early-stage DKD (all P<0.05). Multivariate analysis further identified gender (male, OR = 0.63), age (OR = 1.02), HbA1c (OR = 1.06), GLO (OR = 1.05), TBIL (OR = 0.98), CREA (OR = 1.01), LDL (OR = 0.84), WBC (OR = 1.05), TyG index (OR = 1.39), and NPAR (OR = 1.05) as independent risk factors for early DKD (all P<0.05). Results indicate that male gender, increasing age, poor glycemic control, hyperglobulinemia, elevated creatinine, high white blood cell count, and elevated TyG index are significantly associated with increased risk of early-stage DKD (**Supplementary Figure 1**).

**Table 2:** Univariate and multivariate logistic regression analysis of patients with early diabetic nephropathy and patients with diabetes mellitus alone.

Variables	Univariate					Multivariate				
	β	S.E	Z	*P*	OR (95%CI)	β	S.E	Z	*P*	OR (95%CI)
Gender										
0					1.00 (Reference)					1.00 (Reference)
1	-0.38	0.07	-5.14	**<.001**	0.69 (0.59 ~ 0.79)	-0.46	0.09	-5.26	**<.001**	0.63 (0.53 ~ 0.75)
Age	0.02	0.00	7.85	**<.001**	1.02 (1.02 ~ 1.03)	0.02	0.00	5.05	**<.001**	1.02 (1.01 ~ 1.02)
HBA1c	0.06	0.02	3.84	**<.001**	1.06 (1.03 ~ 1.09)	0.06	0.02	3.35	**<.001**	1.06 (1.02 ~ 1.10)
ALT	-0.00	0.00	-1.65	0.098	1.00 (1.00 ~ 1.00)					
AST	-0.00	0.00	-0.46	0.646	1.00 (1.00 ~ 1.00)					
ALB	-0.03	0.01	-3.83	**<.001**	0.97 (0.95 ~ 0.98)					
GLO	0.07	0.01	8.47	**<.001**	1.07 (1.05 ~ 1.09)	0.05	0.01	5.36	**<.001**	1.05 (1.03 ~ 1.07)
ALP	0.01	0.00	2.13	**0.033**	1.01 (1.01 ~ 1.01)					
TBIL	-0.04	0.01	-5.28	**<.001**	0.96 (0.95 ~ 0.98)	-0.02	0.01	-2.87	**0.004**	0.98 (0.97 ~ 0.99)
DBIL	-0.06	0.02	-2.82	**0.005**	0.94 (0.91 ~ 0.98)					
CREA	0.01	0.00	7.63	**<.001**	1.01 (1.01 ~ 1.01)	0.01	0.00	5.37	**<.001**	1.01 (1.01 ~ 1.01)
UREA	0.10	0.02	6.24	**<.001**	1.11 (1.07 ~ 1.14)					
UA	0.01	0.00	4.38	**<.001**	1.01 (1.01 ~ 1.01)					
TG	0.06	0.02	3.70	**<.001**	1.06 (1.03 ~ 1.09)					
CHOL	-0.03	0.03	-0.89	0.371	0.97 (0.92 ~ 1.03)					
LDL	-0.13	0.04	-3.19	**0.001**	0.88 (0.81 ~ 0.95)	-0.18	0.05	-3.69	**<.001**	0.84 (0.76 ~ 0.92)
HDL	0.02	0.11	0.16	0.876	1.02 (0.83 ~ 1.25)					
GGT	0.00	0.00	0.98	0.328	1.00 (1.00 ~ 1.00)					
GLU	0.04	0.01	4.32	**<.001**	1.04 (1.02 ~ 1.06)					
WBC	0.09	0.02	5.71	**<.001**	1.09 (1.06 ~ 1.13)	0.05	0.02	2.44	**0.015**	1.05 (1.01 ~ 1.09)
RBC	-0.28	0.06	-4.71	**<.001**	0.75 (0.67 ~ 0.85)					
PLT	0.00	0.00	0.52	0.600	1.00 (1.00 ~ 1.00)					
HGB	-0.01	0.00	-5.57	**<.001**	0.99 (0.99 ~ 0.99)					
HCT	-0.04	0.01	-6.12	**<.001**	0.96 (0.94 ~ 0.97)					
MCV	-0.01	0.01	-1.82	0.069	0.99 (0.97 ~ 1.00)					
MCH	-0.03	0.02	-1.55	0.122	0.97 (0.94 ~ 1.01)					
MCHC	0.00	0.00	0.02	0.981	1.00 (0.99 ~ 1.01)					
NEUT per	0.03	0.00	8.00	**<.001**	1.03 (1.02 ~ 1.04)					
LYMPH per	-0.03	0.00	-7.37	**<.001**	0.97 (0.96 ~ 0.98)					
MONO per	-0.04	0.02	-2.30	**0.021**	0.96 (0.93 ~ 0.99)					
EO per	-0.07	0.02	-3.24	**0.001**	0.94 (0.90 ~ 0.97)					
BASO per	-0.66	0.13	-5.03	**<.001**	0.51 (0.40 ~ 0.67)					
NEUT	0.13	0.02	7.04	**<.001**	1.14 (1.10 ~ 1.18)					
LYMPH	-0.19	0.05	-3.57	**<.001**	0.83 (0.75 ~ 0.92)					
MONO	0.52	0.18	2.94	**0.003**	1.68 (1.19 ~ 2.38)					
EO	-0.58	0.31	-1.90	0.057	0.56 (0.31 ~ 1.02)					
BASO	-5.15	1.93	-2.66	**0.008**	0.01 (0.00 ~ 0.26)					
RDW	0.12	0.03	3.53	**<.001**	1.13 (1.05 ~ 1.21)					
TyG	0.18	0.04	4.45	**<.001**	1.20 (1.11 ~ 1.30)	0.33	0.05	6.40	**<.001**	1.39 (1.25 ~ 1.53)
SII	0.01	0.00	4.80	**<.001**	1.01 (1.01 ~ 1.01)					
SIRI	0.08	0.02	4.32	**<.001**	1.08 (1.04 ~ 1.12)					
NLR	0.09	0.02	5.52	**<.001**	1.10 (1.06 ~ 1.13)					
PLR	0.01	0.00	3.96	**<.001**	1.01 (1.01 ~ 1.01)					
NHR	0.08	0.01	6.22	**<.001**	1.09 (1.06 ~ 1.12)					
MHR	0.39	0.12	3.24	**0.001**	1.48 (1.17 ~ 1.88)					
PHR	0.00	0.00	1.86	0.063	1.00 (1.00 ~ 1.00)					
LHR	-0.05	0.04	-1.45	0.147	0.95 (0.89 ~ 1.02)					
NMR	0.04	0.01	4.73	**<.001**	1.04 (1.03 ~ 1.06)					
AISI	0.01	0.00	3.47	**<.001**	1.01 (1.01 ~ 1.01)					
MLR	0.86	0.18	4.65	**<.001**	2.35 (1.64 ~ 3.37)					
NPAR	0.09	0.01	8.32	**<.001**	1.10 (1.07 ~ 1.12)	0.05	0.01	3.74	**<.001**	1.05 (1.02 ~ 1.08)
NHHR	0.01	0.02	0.81	0.417	1.01 (0.98 ~ 1.05)					
UHR	0.01	0.00	3.72	**<.001**	1.01 (1.01 ~ 1.01)					
AIP	0.32	0.10	3.25	**0.001**	1.38 (1.14 ~ 1.68)					
SHR	0.31	0.13	2.34	**0.019**	1.36 (1.05 ~ 1.76)					

OR, Odds Ratio; CI, Confidence Interval. Bold font indicates p-value <0.05.

### 3.3 Comparative performance of machine learning methods

**Figure 2:**
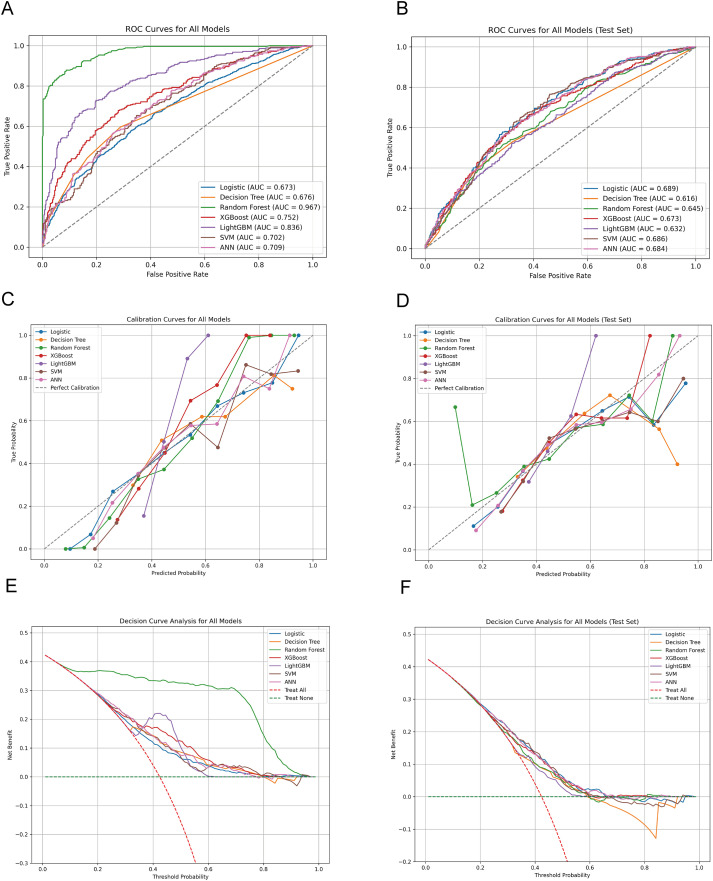
Performance comparison of multiple machine learning models in predicting early diabetic nephropathy **(A)** Receiver operating characteristic (ROC) curves for seven machine learning models on the training set. The area under the curve (AUC) value for each model is annotated next to its respective curve. The dashed grey line represents a classifier with no discriminative power (AUC = 0.5). **(B)** ROC curves for the same models evaluated on the independent test set. **(C)** Calibration curves for the models on the training set. The diagonal dashed line represents perfect calibration, where predicted probabilities perfectly match observed event frequencies. The degree of deviation from this line for each model’s curve reflects its calibration error. **(D)** Calibration curves for the models on the test set. **(E)** Decision curve analysis (DCA) for the models on the training set. The y-axis represents the net benefit calculated across a range of threshold probabilities (x-axis). The grey solid line assumes no patients have the event (‘Treat None’), and the solid black line assumes all patients have the event (‘Treat All’). A model curve higher than these lines indicates clinical utility across that threshold range. **(F)** Decision curve analysis for the models on the test set.

**Supplementary Table 2** summarizes the performance of seven machine learning models on the validation set. The logistic regression model achieved the highest AUC of 0.689, with accuracy of 63.9%, precision of 61.8%, and F1 score of 0.489. While most models exhibited moderate sensitivity (**Figure 2**), LR demonstrated the highest sensitivity (40.5%) among all compared methods, whereas LightGBM achieved the highest specificity (92.1%). All models demonstrated comparable performance in accuracy and precision, yet sensitivity remained relatively low—likely due to the imbalanced nature of the early diabetic nephropathy dataset. Given the clinical priority of identifying early-stage kidney disease cases, we selected the logistic regression model due to its superior discriminative capability (AUC) and relatively higher sensitivity.

### 3.4 Feature importance ranking

**Figure 3:**
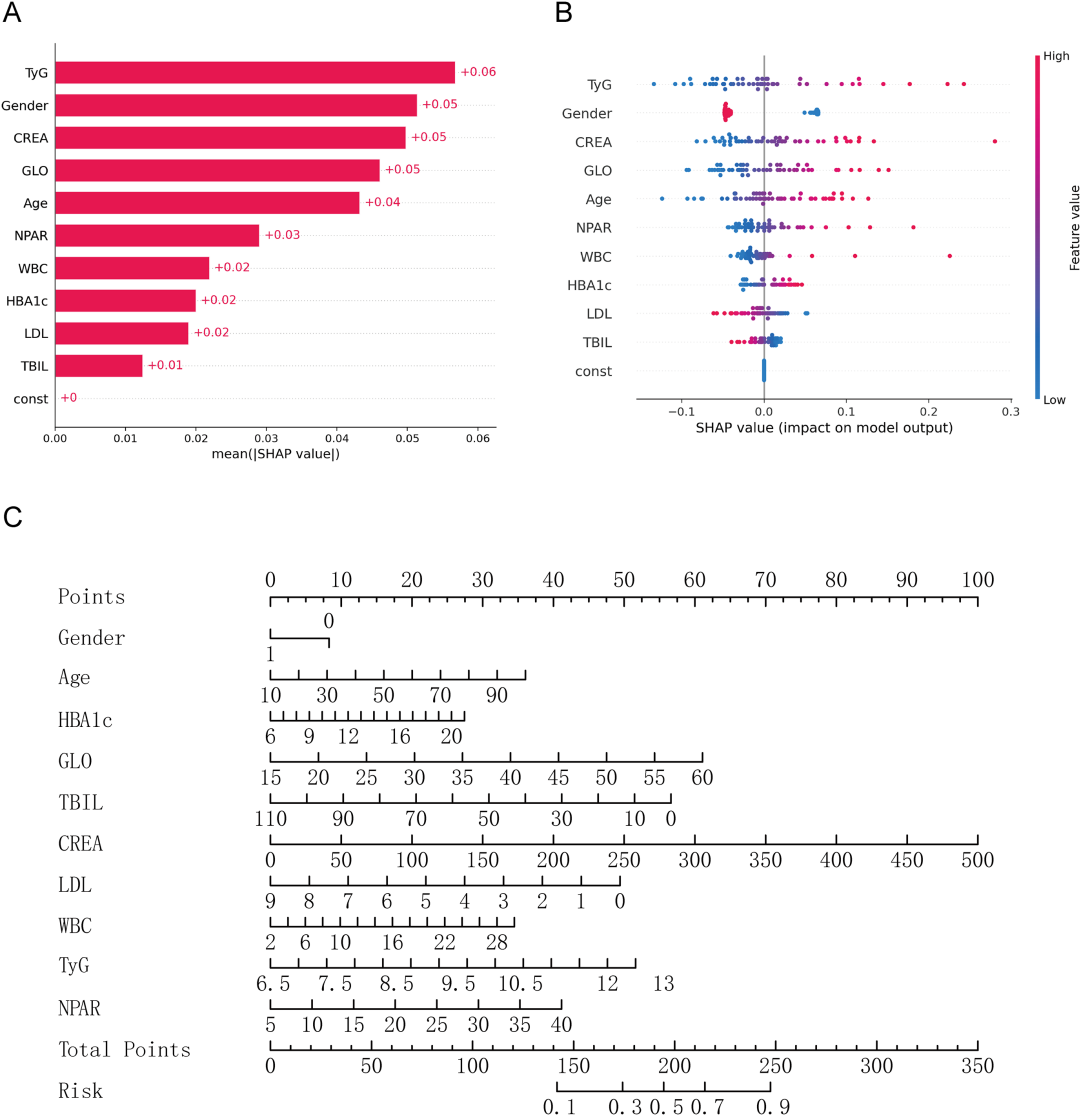
SHAP feature importance analysis and nomogram construction for disease risk prediction model **(A)** SHAP feature importance bar chart, showing the average absolute SHAP value of each feature. Larger values ​​indicate a more significant impact on model predictions. **(B)** SHAP bee swarm plot, showing the distribution of feature values ​​and their corresponding SHAP values ​​for each sample. Color represents high and low feature values ​​(red for high, blue for low), and the degree of horizontal spread reflects the stability of the feature’s influence. **(C)** Logistic regression nomogram, used to calculate total points based on individual feature values ​​(such as gender, age, HBA1c), and map them to risk probabilities, supporting rapid assessment of disease risk in clinical practice.

To determine the characteristics contributing most significantly to predicting early diabetic nephropathy, we ranked their importance based on weights in the logistic regression model (**Figures 3A, B**). The top five variables were TyG, gender, CREA, GLO, and age. Among these, TyG exhibited the highest positive contribution, indicating its crucial role in identifying early nephropathy. Gender, CREA, and GLO demonstrated substantial predictive weights, suggesting that renal function indicators and globulin levels hold key positions in the predictive model. To develop a disease risk prediction tool, we employed logistic regression to construct a nomogram model that quantifies the predictive contribution of each clinical feature (**Figure 3C**).

### 3.5 NHANES database validation

Data from the NHANES database (2005-2018) were selected for external validation. After exclusion, 1,496 diabetic patients remained (**Supplementary Table 3**), with 371 (24.8%) classified into the early diabetic nephropathy group and 1,125 (75.2%) into the simple diabetes group. The mean GLO level was higher in the early diabetic nephropathy group than in the simple diabetes group (31.69 [SD, 4.93] vs 30.64 [SD, 4.57]; P<0.001). Similarly, mean HbA1c (8.54 [SD, 2.08] vs 7.93 [SD, 1.62]; P<0.001), TyG (9.48 [SD, 0.84] vs 9.27 [SD, 0.74]; P<0.001), and NPAR (14.56 [SD, 2.64] vs 14.02 [SD, 2.53]; P<0.001) were significantly higher in the early DKD group.

### 3.6 NHANES database external validation analysis

**Figure 4:**
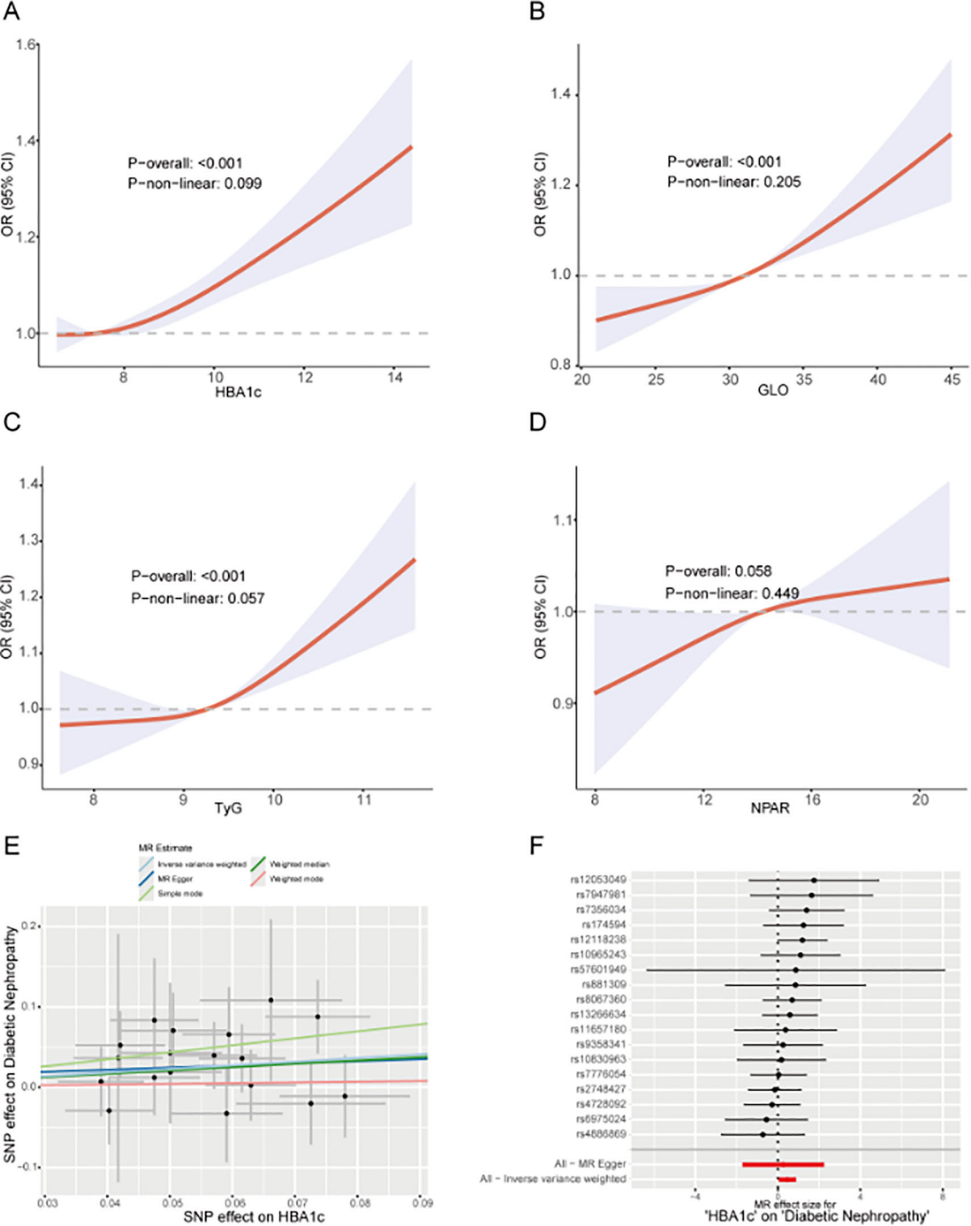
Analysis of the association between key indicators and the risk of diabetic nephropathy **(A)** The RCS curve for HbA1c and diabetic nephropathy risk demonstrates the relationship between OR values (95% CI) and HbA1c levels. P-overall <0.001 indicates overall significance, while P-non-linear = 0.099 indicates non-linearity is not significant. **(B)** RCS curve of GLO versus diabetic nephropathy risk. OR values (95% CI) show a positive correlation. P-overall <0.001 indicates overall significance, while P-non-linear = 0.205 indicates non-linearity is not significant. **(C)** RCS curve of TyG versus diabetic nephropathy risk. OR (95% CI) indicates positive correlation. P-overall <0.001 indicates overall significance. P-non-linear = 0.057 indicates near-significant nonlinearity. **(D)** RCS curve for NPAR and diabetic nephropathy risk. OR (95% CI) shows weak positive correlation, P-overall = 0.058 indicates marginal overall significance, P-non-linear = 0.449 indicates non-linearity is not significant. **(E)** Mendelian randomization scatterplot illustrating the effects of genetic variation (SNP) on HbA1c (exposure) and diabetic nephropathy (outcome). Dotted lines represent fitted curves from different MR methods (e.g., MR Egger), suggesting causal associations. **(F)** Mendelian randomization forest plot listing effect sizes and 95% CIs for each SNP on HbA1c and diabetic nephropathy. The bottom summary line displays the overall causal estimate (e.g., inverse variance weighted), supporting a causal effect of HbA1c.

To investigate independent associations between multiple metabolic indicators and disease risk, univariate and multivariate logistic regression models were employed (**Supplementary Table 4**). In multivariate analysis, HbA1c (OR = 1.14, 95% CI: 1.06–1.22, P<0.001), GLO (OR = 1.05, 95% CI: 1.02–1.07, P<0.001), TyG (OR = 1.27, 95% CI: 1.07–1.51, P = 0.007), and NPAR (OR = 1.08, 95% CI: 1.03–1.14, P<0.001) maintained significant independent associations. Restricted cubic spline analysis revealed nonlinear relationships (**Figures 4A–D**): HbA1c (P-overall <0.001; P-non-linear=0.099), GLO (P-overall <0.001; P-non-linear=0.205), and TyG (P-overall <0.001; P-non-linear=0.057) exhibited significant overall association trends, demonstrating clear dose-response relationships. Mendelian randomization analysis confirmed HbA1c as a risk factor for diabetic nephropathy (**Figures 4E, F**).

## 4 Discussion

This study successfully constructed and validated a machine learning predictive model for early diabetic kidney disease based on routine blood and biochemical indicators. By comparing seven machine learning algorithms, logistic regression was selected as the optimal model (AUC = 0.689) and underwent external validation in the NHANES database. Glycated hemoglobin (HbA1c), globulin (GLO), and the triglyceride-glucose index (TyG) were identified as the most predictive factors. Mendelian randomization analysis further provided genetic evidence for a causal relationship between HbA1c and DKD.

Compared to previous applications of machine learning in diabetes diagnosis, this study extends the predictive target to identification of early-stage DKD (17, 18). Although ensemble algorithms like XGBoost and LightGBM demonstrate superior performance in certain medical prediction tasks, they did not surpass LR performance in this study (19, 20). This phenomenon may be attributed to the scarcity of early-stage DKD samples and significant class imbalance, which makes complex ensemble algorithms more prone to overfitting on the majority class (21). The LR model, leveraging its inherent regularization mechanism and linear assumption, demonstrates superior generalization capability under limited sample conditions (22).

The model’s relatively low sensitivity (40.5%) reflects the inherent diagnostic challenges of early-stage DKD. Pathological alterations in early-stage DKD are relatively subtle, with limited variations in conventional blood markers. Combined with the heterogeneity of disease progression, this makes it difficult for prediction models based on traditional biochemical indicators to achieve ideal sensitivity. However, considering this model is primarily positioned as a primary screening tool, its high specificity (81.3%) helps reduce false-positive results, thereby avoiding unnecessary consumption of medical resources and patient anxiety.

The TyG index ranked first in feature importance, underscoring the central role of insulin resistance in DKD pathogenesis. Insulin resistance accelerates glomerulosclerosis and interstitial fibrosis through multiple mechanisms, including activation of the renin-angiotensin-aldosterone system, promotion of advanced glycation end-product formation, and induction of oxidative stress and inflammatory responses (23, 24). As a convenient assessment tool for insulin resistance, the TyG index’s prominent position in DKD prediction provides a clear target for early clinical intervention.

The inclusion of CREA and GLO as traditional markers of renal function and inflammation in the model has solid theoretical foundation (25). Notably, even in early-stage DKD patients with eGFR ≥60 ml/min/1.73 m², subtle changes in CREA retain predictive value, suggesting that serum creatinine’s sensitivity in assessing early renal impairment may be underestimated (26). Elevated GLO reflects chronic systemic inflammation, a key driver of DKD progression (27). The inclusion of the novel indicator NPAR further supports the role of the “inflammation-malnutrition-atherosclerosis” syndrome in DKD pathogenesis (28). To assess multicollinearity, variance inflation factors (VIF) were calculated for all predictors. While moderate collinearity was observed between TyG and its component variables (TG, glucose), no features exceeded the VIF>10 threshold for exclusion. Comparative analysis of base laboratory values alone, composite indices alone, and combined features demonstrated that the combined approach achieved optimal predictive performance, though a VIF-pruned model offered comparable results with improved coefficient stability (**Supplementary Table 7**).

External validation using the NHANES database significantly enhances study reliability. Validation results demonstrate that key predictors (HbA1c, GLO, TyG, NPAR) retain substantial predictive value in an independent U.S. population, confirming the cross-population consistency of these biomarkers. Restricted cubic spline analysis revealed dose-response relationships between HbA1c, GLO, and TyG with DKD risk, providing a refined basis for clinical risk stratification. However, the weakened predictive effects of certain traditional indicators in external validation suggest the influence of population heterogeneity. Differences in genetic backgrounds, lifestyles, and healthcare systems between Chinese and American populations may influence biomarker expression and predictive value (29, 30).

Mendelian randomization analysis provides robust genetic evidence for the causal relationship between HbA1c and DKD, overcoming limitations of observational studies such as confounding factors and reverse causality (31). This finding validates the fundamental role of strict glycemic control in primary prevention of DKD and provides theoretical support for the critical role of HbA1c in our predictive model (32).

The nomogram developed in this study provides clinicians with an intuitive and user-friendly risk assessment tool (33). Predictive models based on routine examination indicators offer advantages of high cost-effectiveness and strong accessibility, making them particularly suitable for implementation in primary care settings and resource-limited regions (34). Clinicians can rapidly identify individuals at high risk for DKD based on patients’ routine laboratory results, thereby implementing targeted monitoring and intervention strategies.

This study has several limitations: First, the single-center retrospective design limits research representativeness; a multicenter prospective study would better validate model generalizability. Second, the relatively scarce early-stage DKD samples may cause category imbalance affecting model performance; future studies may consider optimizing training strategies using methods such as Synthetic Minority Over-sampling Technique (SMOTE). Third, cross-sectional studies cannot assess the impact of dynamic changes on predictive accuracy; longitudinal cohort studies would provide more comprehensive risk assessment. Fourth, the absence of kidney biopsy confirmation means that some patients classified as early DKD may have had CKD from other etiologies. This diagnostic ambiguity, inherent to clinical practice, may contribute to the model’s moderate sensitivity. However, this reflects real-world screening scenarios where biopsy is rarely performed for early-stage kidney disease. Finally, the model did not incorporate kidney-specific biomarkers or genetic risk scores, which may limit further improvements in discriminatory performance.

## 5 Conclusions

This study successfully developed a machine learning model based on routine blood and biochemical indicators for prediction and risk stratification of early diabetic kidney disease. Key predictors such as HbA1c, GLO, and TyG were identified, providing valuable methodological contributions for non-invasive, low-cost screening of early DKD. This approach holds promise for playing a significant role in clinical practice, particularly in primary care settings and resource-limited environments where comprehensive kidney function assessment may not be readily available.

## Data Availability

The original contributions presented in the study are included in the article/**Supplementary Material**. Further inquiries can be directed to the corresponding authors.
