# Supplementary material for: Machine learning model based on routine blood and biochemical parameters for early diagnosis of diabetic kidney disease
Source: Front Endocrinol (Lausanne). 2026 Jan 28;17:1720574. doi: 10.3389/fendo.2026.1720574 (PMC12890677; doi:10.3389/fendo.2026.1720574)
Supplement: Supplementary file 1 [file DataSheet1.docx]

**Supplementary** **Table 1**

Supplementary Table 1. Calculation formula of each index

| Index | English Name | Calculation Formula |
| --- | --- | --- |
| TyG | Triglyceride-Glucose Index | ln[ fasting TG (mg/dL) × fasting glucose (mg/dL) / 2 ] |
| SII | Systemic Immune-Inflammation Index | (neutrophil count × platelet count) / lymphocyte count (units usually 10⁹/L) |
| SIRI | Systemic Inflammation Response Index | (neutrophil count × monocyte count) / lymphocyte count (units usually 10⁹/L) |
| NLR | Neutrophil-to-Lymphocyte Ratio | neutrophil count (10⁹/L) / lymphocyte count (10⁹/L) |
| PLR | Platelet-to-Lymphocyte Ratio | platelet count (10⁹/L) / lymphocyte count (10⁹/L) |
| PNR | Patient Nutrition Risk Index (same formula as PNI) | serum albumin (g/L) + 5 × lymphocyte count (×10⁹/L) |
| NHR | Neutrophil-to-HDL-Cholesterol Ratio | neutrophil count (10⁹/L) / HDL-C (mmol/L or mg/dL) |
| MHR | Monocyte-to-HDL-Cholesterol Ratio | monocyte count (10⁹/L) / HDL-C (mmol/L or mg/dL) |
| PHR | Platelet-to-HDL-Cholesterol Ratio | platelet count (10⁹/L) / HDL-C (mmol/L or mg/dL) |
| LHR | Lymphocyte-to-HDL-Cholesterol Ratio | lymphocyte count (10⁹/L) / HDL-C (mmol/L or mg/dL) |
| NMR | Neutrophil-to-Monocyte Ratio | neutrophil count (10⁹/L) / monocyte count (10⁹/L) |
| AISI | Aggregate Index of Systemic Inflammation | (platelet count × neutrophil count × monocyte count) / lymphocyte count (units usually 10⁹/L) |
| MLR | Monocyte-to-Lymphocyte Ratio | monocyte count (10⁹/L) / lymphocyte count (10⁹/L) |
| NPAR | Neutrophil Percentage-to-Albumin Ratio | (neutrophil percentage (%) × 100) / albumin (g/dL) |
| NHHR | Non-HDL-Cholesterol-to-HDL-Cholesterol Ratio | (total cholesterol – HDL-C) / HDL-C  or non-HDL-C / HDL-C |
| UHR | Uric-acid-to-HDL-Cholesterol Ratio | serum uric acid (µmol/L or mg/dL) / HDL-C (mmol/L or mg/dL) |
| AIP | Atherogenic Index of Plasma | log [ TG (mmol/L) / HDL-C (mmol/L) ] |
| SHR | Stress Hyperglycemia Ratio | FPG / (1.59 × HbA1c – 2.59) |
| RA | RDW-to-Albumin Ratio | RDW (%) / albumin (g/dL) |
| PNI | Prognostic Nutritional Index | serum albumin (g/L) + 5 × lymphocyte count (×10⁹/L) |

**Supplementary Table 2**

Supplementary Table 2 Comparison of performance of seven machine learning methods

| Model | AUC | Accuracy | Precision | Sensitivity | Specificity | Balanced  accuracy | F1 Score |
| --- | --- | --- | --- | --- | --- | --- | --- |
| Logistic | 0.689 | 0.639 | 0.618 | 0.405 | 0.813 | 0.609 | 0.489 |
| Decision Tree | 0.616 | 0.624 | 0.619 | 0.313 | 0.856 | 0.584 | 0.415 |
| Random Forest | 0.645 | 0.627 | 0.588 | 0.425 | 0.778 | 0.601 | 0.493 |
| XGBoost | 0.673 | 0.631 | 0.632 | 0.330 | 0.856 | 0.593 | 0.433 |
| LightGBM | 0.632 | 0.603 | 0.628 | 0.178 | 0.921 | 0.549 | 0.277 |
| SVM | 0.686 | 0.633 | 0.609 | 0.398 | 0.809 | 0.603 | 0.481 |
| ANN | 0.684 | 0.632 | 0.609 | 0.390 | 0.813 | 0.602 | 0.476 |

**Supplementary Table 3**

Supplementary Table 3: Characteristics of Patients with Early Diabetic Nephropathy and Patients with Diabetes Mellitus Alone in the NHANES Database, 2005–2018

| Variables | Total  (n = 1496) | Diabetes  (n = 1125) | Diabetic  Nephropathy  (n = 371) | Statistic | *P* |
| --- | --- | --- | --- | --- | --- |
|  |  |  |  |  |  |
| Age, Mean ± SD | 58.14 ± 13.59 | 57.77 ± 13.52 | 59.26 ± 13.79 | t=-1.83 | 0.067 |
| CREA, Mean ± SD | 0.80 ± 0.19 | 0.80 ± 0.19 | 0.80 ± 0.19 | t=0.08 | 0.938 |
| TBIL, Mean ± SD | 0.65 ± 0.29 | 0.65 ± 0.29 | 0.65 ± 0.29 | t=0.03 | 0.978 |
| GLO, Mean ± SD | 30.90 ± 4.68 | 30.64 ± 4.57 | 31.69 ± 4.93 | t=-3.77 | **<.001** |
| GHB, Mean ± SD | 8.08 ± 1.76 | 7.93 ± 1.62 | 8.54 ± 2.08 | t=-5.14 | **<.001** |
| TyG, Mean ± SD | 9.32 ± 0.77 | 9.27 ± 0.74 | 9.48 ± 0.84 | t=-4.42 | **<.001** |
| NPAR, Mean ± SD | 14.16 ± 2.57 | 14.02 ± 2.53 | 14.56 ± 2.64 | t=-3.50 | **<.001** |
| Gender, n(%) |  |  |  | χ²=0.88 | 0.349 |
| Female | 713 (47.66) | 544 (48.36) | 169 (45.55) |  |  |
| Male | 783 (52.34) | 581 (51.64) | 202 (54.45) |  |  |
| t: t-test, χ²: Chi-square test | | | | | |
| SD: standard deviation | | | | | |

**Supplementary Table 4**

Supplementary Table 4: Univariate and Multivariate Logistic Regression

| Variables | Univariate | | | | |  | Multivariate | | | | |
| --- | --- | --- | --- | --- | --- | --- | --- | --- | --- | --- | --- |
|  | β | S.E | Z | *P* | OR (95%CI) |  | β | S.E | Z | *P* | OR (95%CI) |
| Gender |  |  |  |  |  |  |  |  |  |  |  |
| 1 |  |  |  |  | 1.00 (Reference) |  |  |  |  |  |  |
| 2 | -0.11 | 0.12 | -0.94 | 0.349 | 0.89 (0.71 ~ 1.13) |  |  |  |  |  |  |
| Age | 0.01 | 0.00 | 1.83 | 0.067 | 1.01 (1.00 ~ 1.02) |  |  |  |  |  |  |
| GHB | 0.18 | 0.03 | 5.64 | **<.001** | 1.20 (1.12 ~ 1.27) |  | 0.13 | 0.04 | 3.55 | **<.001** | 1.14 (1.06 ~ 1.22) |
| GLO | 0.05 | 0.01 | 3.72 | **<.001** | 1.05 (1.02 ~ 1.07) |  | 0.05 | 0.01 | 3.51 | **<.001** | 1.05 (1.02 ~ 1.07) |
| TBIL | -0.01 | 0.21 | -0.03 | 0.978 | 0.99 (0.66 ~ 1.49) |  |  |  |  |  |  |
| CREA | -0.02 | 0.31 | -0.08 | 0.938 | 0.98 (0.53 ~ 1.79) |  |  |  |  |  |  |
| TyG | 0.36 | 0.08 | 4.64 | **<.001** | 1.43 (1.23 ~ 1.67) |  | 0.24 | 0.09 | 2.70 | **0.007** | 1.27 (1.07 ~ 1.51) |
| NPAR | 0.08 | 0.02 | 3.48 | **<.001** | 1.09 (1.04 ~ 1.14) |  | 0.08 | 0.02 | 3.38 | **<.001** | 1.08 (1.03 ~ 1.14) |
| OR: Odds Ratio, CI: Confidence Interval | | | | | | | | | | | |

**Supplementary Table** **5**

Supplementary Table S5: Diagnostic Criteria and Exclusion Process

| **Criteria** | **Description** |
| --- | --- |
| **Inclusion Criteria** |  |
| Diabetes diagnosis | HbA1c ≥6.5% (WHO criteria) |
| Early DKD definition | UACR 30-300 mg/g AND eGFR ≥60 ml/min/1.73m² |
| Simple diabetes | UACR <30 mg/g AND eGFR ≥60 ml/min/1.73m² |
| **Exclusion Criteria** |  |
| Non-diabetic kidney disease | Documented glomerulonephritis, polycystic kidney disease, obstructive uropathy, or kidney stones |
| Acute kidney injury | Rapid eGFR decline >25% within 3 months |
| Urinary tract infection | Active UTI at time of UACR measurement |
| Recent contrast exposure | Within 72 hours of laboratory testing |
| Missing essential data | Incomplete UACR, eGFR, or HbA1c values |

**Supplementary Table 6**

Supplementary Table 6: Hyperparameters for All Models

| **Model** | **Hyperparameter** | **Final Value** | **Search Range** |
| --- | --- | --- | --- |
| **Logistic Regression** |  |  |  |
|  | Regularization (C) | 1.0 | [0.001, 0.01, 0.1, 1, 10, 100] |
|  | Penalty | L2 | [L1, L2, ElasticNet] |
|  | Solver | lbfgs | [lbfgs, saga] |
|  | Class weight | balanced | [None, balanced] |
| **Decision Tree** |  |  |  |
|  | Max depth | 8 | [3, 5, 8, 10, 15, None] |
|  | Min samples split | 20 | [2, 5, 10, 20, 50] |
|  | Min samples leaf | 10 | [1, 5, 10, 20] |
|  | Criterion | gini | [gini, entropy] |
| **Random Forest** |  |  |  |
|  | n_estimators | 200 | [50, 100, 200, 500] |
|  | Max depth | 10 | [5, 10, 15, 20, None] |
|  | Min samples split | 10 | [2, 5, 10, 20] |
|  | Max features | sqrt | [sqrt, log2, 0.5] |
| **SVM** |  |  |  |
|  | Kernel | RBF | [linear, RBF, poly] |
|  | C | 1.0 | [0.1, 1, 10, 100] |
|  | Gamma | scale | [scale, auto, 0.01, 0.1] |
| **XGBoost** |  |  |  |
|  | n_estimators | 150 | [50, 100, 150, 200, 300] |
|  | Max depth | 5 | [3, 5, 7, 9] |
|  | Learning rate | 0.1 | [0.01, 0.05, 0.1, 0.2] |
|  | Subsample | 0.8 | [0.6, 0.8, 1.0] |
|  | Colsample_bytree | 0.8 | [0.6, 0.8, 1.0] |
| **LightGBM** |  |  |  |
|  | n_estimators | 200 | [50, 100, 200, 300] |
|  | Max depth | 6 | [4, 6, 8, 10, -1] |
|  | Learning rate | 0.05 | [0.01, 0.05, 0.1] |
|  | Num leaves | 31 | [15, 31, 63, 127] |
| **DNN** | See detailed description below |  |  |

**Supplementary Table 7**

Supplementary Table 7: VIF for all Features in the Full Model

| **Feature** | **VIF** | **Interpretation** |
| --- | --- | --- |
| TyG | 8.42 | Moderate collinearity |
| GLU | 7.89 | Moderate collinearity |
| TG | 6.73 | Moderate collinearity |
| AIP | 5.21 | Moderate collinearity |
| HDL-C | 4.87 | Acceptable |
| NPAR | 4.12 | Acceptable |
| Other features | <4 | Acceptable |

**Supplementary Figure 1**

**
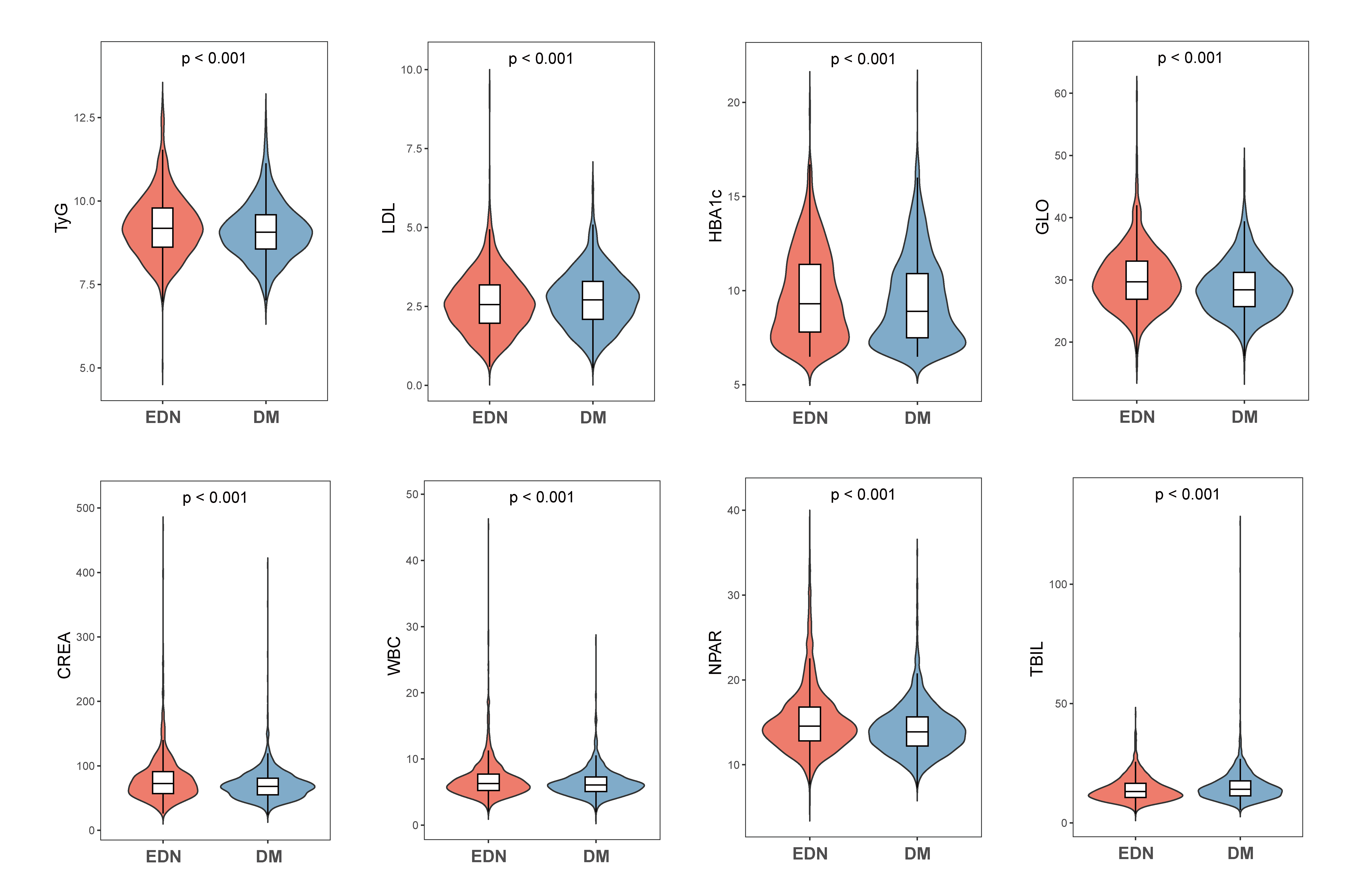
**

**Supplementary Figure 1.** **Boxplot Comparing Distribution of 8 Biomarkers Between Early Diabetic Nephropathy and Diabetes Mellitus Groups**

**A-H:** Boxplot showing TyG index, LDL, HBA1c, GLO, CREA, WBC, NPAR, TBIL distribution in EDN (left) and DM (right) groups, visualizing median and variability, p < 0.001 indicates a significant difference between groups.

**Supplementary Figure 2**


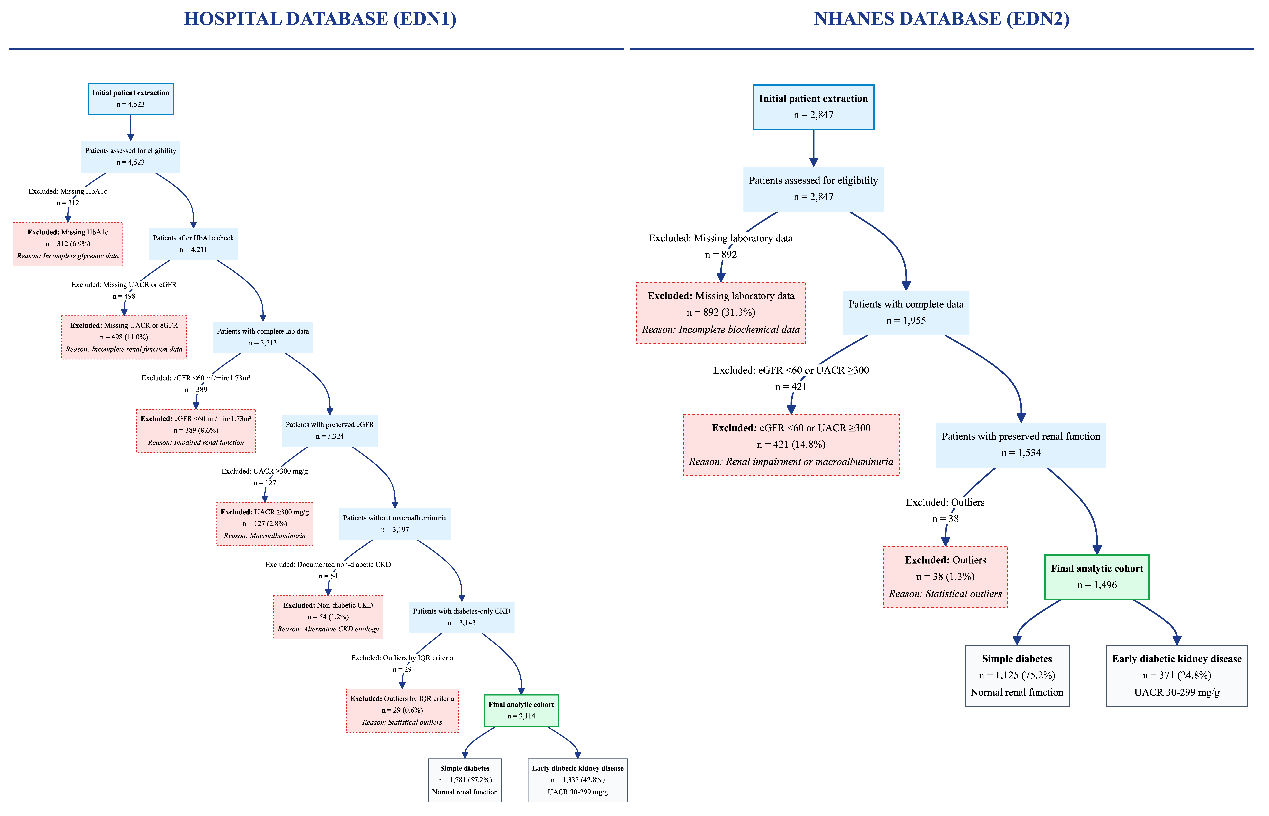


**Supplementary Figure 2.** Flowchart of participant selection
